# The Distribution of Fat and Strychnine in Nux Vomica Seeds

**Published:** 1904-09-10

**Authors:** 


### THE DISTRIBUTION OF FAT AND STRYCHNINE IN NUX VOMICA SEEDS.

In a paper read before the British Pharmaceutical Conference at Sheffield, Messrs. H. Wippell Gadd and Sydney C. Gadd called attention to some experimental work which they had carried out with a view to obviate the difficulties which are experienced in the manufacture of the official liquid extract of nux vomica on account of the fat which the seeds contain.

After steaming a quantity of nux vomica seeds to soften them, they passed them through a Carter's disintegrator. The centrifugal action of the disintegrator separated the light hairs from the heavier particles which make up the bulk of the seeds, and it was thought that it might be advantageous to reject these hairs, as they probably contained more fat than did the remainder of the seeds.

Before doing so, samples of the hairs (which [illegible]mounted to about 5 per cent. of the whole) and of the [illegible] rest of the powder were taken and examined by [illegible] following methods: (1) A portion of each [illegible]ple was freed from fat by prolonged treatment in [illegible]xhlet tube with boiling ether, with the following [illegible]lts:—

| | |
|---|---|
| [illegible]ercentage of fat found in hairs ... ... | 7 |
| [illegible]ercentage of fat found in remainder of the seeds ... ... ... ... ... ... | 2 |
| [illegible]ercentage of fat in whole seeds (by calculation) ... ... ... ... ... ... | 2·25 |

[illegible]nother portion of each sample was examined for [illegible]nine by Bird's process[1] with the following [illegible]:—

| | |
|---|---|
| [illegible]entage of strychnine in hairs ... ... | 0 5 |
| [illegible]ntage of strychnine in remainder of [illegible]ds ... ... ... ... ... ... | 1·5 |
| [illegible]ntage of strychnine in whole seeds (by [illegible]ulation) ... ... ... ... ... ... | 1·4 |

[illegible]hese results it is obvious that by rejecting [illegible] a considerable portion of the fat would be [illegible] whilst the loss of strychnine would be immaterial. A liquid extract was therefore made from the seeds from which the hairs had been removed. The liquid extract was made by the official process, but the percolation was continued until the product ceased to give a precipitate with the usual alkaloidal reagents. The weaker percolates were concentrated, and the finished product adjusted to the strength of 1·5 per cent. The amount of fat in the liquid extract was then estimated by the following method: The extract was evaporated almost to dryness, and the residue ground with powdered glass. The fat was extracted by treating the mixture with boiling ether in a Soxhlet tube, with the following result:—

Percentage of fat in liquid extract ... ... 0·62

It was therefore evident that the amount of alcohol (70 per cent), which was sufficient to extract the whole of the strychnine present in the seeds (freed from hairs), only removed one-third of the fat. The liquid extract made thus was of excellent quality and free from any objectionable amount of fat. Further experiments showed that the skins of the seeds contain very little fat, and therefore there is no need to reject the skins as well as the hairs.

Summing up, the authors state that (1) the hairs of nux vomica seeds contain proportionately much more fat and less strychnine than do the inner portion of the seeds, and it follows that (2) the hairs can be rejected with advantage before making the liquid extract.

[1] Pharmaceutical Jour., 1900, vol. ii., p. 574.

